# Brain atrophy pattern in patients with mild cognitive impairment: MRI study

**DOI:** 10.1515/tnsci-2022-0248

**Published:** 2022-09-27

**Authors:** Rosalinda Calandrelli, Marco Panfili, Valeria Onofrj, Huong Elena Tran, Francesca Piludu, Valeria Guglielmi, Cesare Colosimo, Fabio Pilato

**Affiliations:** Dipartimento di Diagnostica per Immagini, Radioterapia, Oncologia ed Ematologia, Institute of Radiology, Fondazione Policlinico Universitario Agostino Gemelli IRCCS, Largo A. Gemelli, 1, 00168 Rome, Italy; Department of Medical Imaging, Cliniques Universitaires Saint-Luc, Brussels, Belgium; Fondazione Policlinico Universitario Agostino Gemelli IRCCS, Largo A. Gemelli, 1, 00168 Rome, Italy; Department of Radiology and Diagnostic Imaging, IRCCS Regina Elena National Cancer Institute, Via Elio Chianesi 53, 00144 Rome, Italy; Institute of Neurology, Fondazione Policlinico Universitario Agostino Gemelli IRCCS, Largo A. Gemelli, 1, 00168 Rome, Italy; Department of Medicine, Unit of Neurology, Neurophysiology, Neurobiology, Campus Bio-Medico University, Rome 00128, Italy

**Keywords:** brain MRI, regional atrophy patterns, visual rating scales, regional thickness and volume measures

## Abstract

We evaluated the accuracy of the quantitative and semiquantitative analysis in detecting regional atrophy patterns and differentiating mild cognitive impairment patients who remain stable (aMCI-S) from patients who develop Alzheimer’s disease (aMCI-AD) at clinical follow-up. Baseline magnetic resonance imaging was used for quantitative and semiquantitative analysis using visual rating scales. Visual rating scores were related to gray matter thicknesses or volume measures of some structures belonging to the same brain regions. Receiver operating characteristic (ROC) analysis was performed to assess measures’ accuracy in differentiating aMCI-S from aMCI-AD. Comparing aMCI-S and aMCI-AD patients, significant differences were found for specific rating scales, for cortical thickness belonging to the middle temporal lobe (MTL), anterior temporal (AT), and fronto-insular (FI) regions, for gray matter volumes belonging to MTL and AT regions. ROC curve analysis showed that middle temporal atrophy, AT, and FI visual scales showed better diagnostic accuracy than quantitative measures also when thickness measures were combined with hippocampal volumes. Semiquantitative evaluation, performed by trained observers, is a fast and reliable tool in differentiating, at the early stage of disease, aMCI patients that remain stable from those patients that may progress to AD since visual rating scales may be informative both about early hippocampal volume loss and cortical thickness reduction.

## 1 Introduction

Dementia is a clinical syndrome caused by various brain diseases and Alzheimer’s disease (AD) is the most frequent [1]. Mild cognitive impairment (MCI) is a predementia stage and it is known that the progression rate to dementia is approximately 15% per year [2]. Subjects with the amnesic subtype of MCI (aMCI) have a higher risk to progress to AD dementia [3–5] although a substantial proportion of aMCI subjects remains stable for years or revert to normal. These observations indicate that clinical aMCI symptoms can also stem from non-AD-related etiologies [6], making patients with aMCI a challenge in the clinical setting [7].

Structural magnetic resonance imaging (MRI), positron emission tomography, cerebrospinal fluid (CSF), or neurophysiological biomarkers have been applied to narrow the differential diagnosis and assess the risk of conversion to dementia [8–11]. To date, several neuroimaging studies assessed atrophy patterns using simple visual rating scales or more complex quantitative manual or automated techniques in MCI patients [12–15]. Visual rating scales provide semiquantitative measures of the degree of atrophy in sectorized brain regions, evaluating structures and liquor spaces of one or more neighboring lobes [16,17]. They do not require dedicated software, are quick to apply, and are designed specifically for routine MRI studies [17], but they can lead to ambiguity and variability among observers [18,19]. On the other hand, automatic volumetric techniques perform the analysis of both gray matter (GM) volume/thickness, by using a surface morphometry approach, and subcortical structure volume, by voxel-based morphometry approach [20]. However, quantitative measures need sophisticated post-processing and mathematical algorithms, as well as a learning MR dataset to assess automated diagnostic results and automated commercial tools [21].

Some quantitative studies have adopted morphometric data such as GM volume and cortical thickness to detect the atrophy of middle temporal lobe (MTL) regions in MCI [22]. Regional thickness measurements are often preferred to volume measurements probably because thickness measures are less influenced by the anatomical variability of the cortex defined by the folding and branching patterns of the collateral sulcus, which dramatically affects the location of the borders [23]. Moreover, in early stage, MCI cortical thinning is more prominent than GM volume loss that follows at the latter stage of the disease [22]. Although some studies did not find a significant volume or thickness loss of specific subregions of MTL at the MCI stage [23], other studies found that combined data of cortical thickness and volume measures in some subregions of MTL may better predict the conversion of MCI to AD compared to estimates of single specific cortical subregions [24]. Moreover, the assessment of only MTL may be unsatisfactory to suggest underlying AD pathology because other regions of the fronto-parieto-temporal network are involved in this pathologic process [25,26].

In aMCI, we hypothesized that an accurate characterization of combined cortical thickness and subcortical volume measures in brain regions matching regions detected on visual rating scale may enhance the predictivity of the future conversion to AD.

Objectives of the current study were (1) to evaluate, in aMCI patients, the relationship between each validated visual rating scale and a set of thickness/volume measurements in matching regions, (2) to evaluate the accuracy of the regional quantitative and semiquantitative analysis in detecting regional patterns of atrophy and their usefulness in differentiating aMCI patients either who may remain stable (aMCI-S) or progress towards AD (aMCI-AD), and (3) to evaluate whether combined visual rating scores or corresponding quantitative measures belonging to matching atrophic regions may increase the accuracy in differentiating aMCI-AD from aMCI-S.

## 2 Materials and methods

### 2.1 Study population

Among patients referred to the Memory Clinic of the Policlinico Universitario “A. Gemelli” in Rome, a total of 300 patients had a full clinical workup and the diagnosis of aMCI and they performed brain MRI. All subjects were native Italian speakers and none of them had a history of traumatic head injury, alcoholism, epilepsy, or stroke, nor other relevant neurologic, psychiatric, and general medical diseases.

Clinical evaluation included medical history, physical and neurological examination, an extensive neuropsychological evaluation including Mini-Mental State Evaluation (MMSE), functional evaluation by clinical dementia rating scale (CDRS), and activity daily living scales.

aMCI was diagnosed according to the current clinical criteria [27] and they were clinically evaluated after 24 months (i.e., 2 years). The diagnosis of AD was based on the current clinical criteria when CDRS was >1 and when patient showed functional impairment [28].

### 2.2 Groups stratification

Based on the clinical evaluations performed after 2 years from diagnosis of aMCI, patients were divided into two sub-groups: stable aMCI (aMCI-S) and progressive aMCI (aMCI-AD). We defined aMCI-S when neurologic evaluation at follow-up visit was unchanged and aMCI-AD when they fulfilled AD criteria.

At follow-up, patients showing symptoms or signs of other dementia syndromes such as frontotemporal dementia, Lewy body dementia, and vascular-ischemic dementia were excluded from the current analysis to ensure a clinically homogeneous group.

Each group of patients was compared with an age-matched group of healthy subjects who had undergone brain MRI scans for various incidental reasons, including trauma, headache, and whose brain MRIs were unremarkable.

All subjects were right-handed, according to the Handedness Questionnaire [29].


**Ethical approval:** The research related to human use has been complied with all the relevant national regulations, institutional policies and in accordance the tenets of the Helsinki Declaration, and has been approved by the authors' institutional review board or equivalent committee. 
**Informed consent:** Informed consent has been obtained from all individuals included in this study. 

### 2.3 Structural MRI

We analyzed MRI images acquired at the time of aMCI diagnosis (Time 0) for the two groups of patients (aMCI-S and aMCI-AD) to detect the presence of some qualitative and quantitative differences that preceded the clinical manifestations of AD occurring in the following 2 years’ time.

All participants underwent brain MR with a 1.5T PHILIPS Ingenia Scanner (Philips Healthcare, Eindhoven, The Netherlands) with a dedicated protocol including a 3D T1-weighted gradient echo sequence (3D-TFE), sagittal, axial and coronal 2D-T2 weighted images, SWAN, and FLAIR images.

Sagittal 3D T1-weighted turbo field eco sequence (3D-T1 W-TFE) was used for thickness/volume quantitative analysis (150 slices with TR = 9.8 ms, TE = 4.6 ms, in plane resolution = 1.0 mm^2^ × 1.0 mm^2^, slice thickness = 1.0 mm, flip angle *a* = 10°, FOV = 200 mm × 222 mm, acquisition matrix = 200 × 222, NSA = 2) and for semiquantitative analysis by visual rating scales.

### 2.4 Semiquantitative analysis of brain atrophy by visual rating scales

MR images were scored by visual rating scales.

Visual rating of all aMCI patients was performed by two examiners with 14-year experience in neuroradiology, blinded to all clinical and pathological information except the person’s age at the time of scanning, according to some previous studies [30–32]. Disagreements were resolved by consensus.

Regional atrophy patterns were rated based on currently used scales: (i) the five-point anterior temporal scale (AT) by Davies et al. [33] and Kipps et al. [34], (ii) the five-point medial temporal lobe atrophy scale (MTA) by Scheltens et al. [18], and (iii) the four-point posterior atrophy scale (PA) by Koedam et al. [32].

AT defines, for each side, the atrophy of part of the frontal and temporal lobe and evaluates the region that connects the frontal lobe to the temporal fronto-insula rating; MTA defines the atrophy of each temporal lobe and PA defines atrophy of both parietal lobes and cuneus gyri of occipital lobes.

To provide an additional, more fine-grained assessment of anterior atrophy other three regional scales were evaluated: orbito-frontal (OF), anterior cingulate (AC), and fronto-insula (FI) scales [35–37]. For OF, AC, and FI scales, a four-part grading system was used; only for the FI scale, separate scores for left and right sides were recorded.

### 2.5 Quantitative analysis of brain atrophy by thickness and volume measurements

Cortical, subcortical, and deep GM structures were processed and segmented using Freesurfer image analysis software (version 6.0.0), which is documented and freely available for download online (http://surfer.nmr.mgh.harvard.edu/) [20,38–41].

Briefly, the processing pipeline includes motion correction and averaging [42] of volumetric T1-weighted images, removal of non-brain tissue [39], automated Talairach transformation, segmentation of the subcortical white matter (WM) and deep GM volumetric structures (including the hippocampus, amygdala, caudate, putamen, and ventricles) [40,43], intensity normalization [44], tessellation of the GM/WM boundary, automated topology correction [45,46], and surface deformation following intensity gradients to place the GM/WM and GM/CSF borders optimally [20,47,48]. Cortical thickness was measured as the closest distance from the GM/WM boundary to the GM/CSF boundary at each vertex. Cortical parcellations were made according to Desikan atlas [41] and thickness/volume measurements were collected from bilateral regions of interest [49].

**Figure 1:**
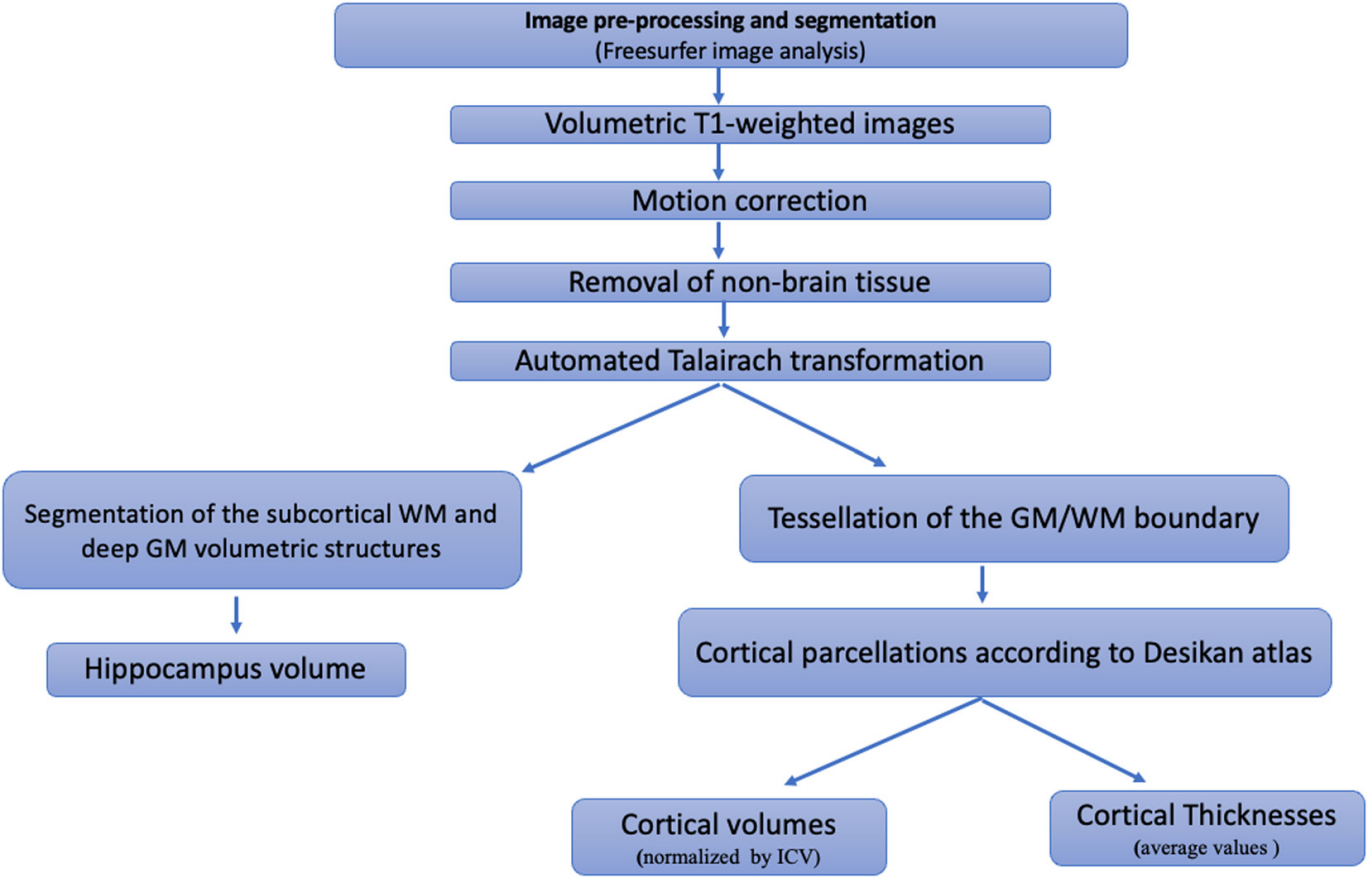
A representative image segmentation flowchart using Freesurfer image analysis software and selection of the structures used to differentiate aMCI-AD from aMCI-S. WM, white matter; GM, gray matter.

Among the multitude of data processed by Freesurfer image analysis software, only the cortical volumes, the subcortical volume of hippocampi, and the cortical thickness measurements were selected, because they are the main structures involved in MCI [7,50] (Figure 1).

All volumetric measurements were normalized for head size by including total intracranial volume while for thickness measurements, the average value was computed.

### 2.6 Relationship between visual rating scales and quantitative regional measures

The visual rating score of each region was compared to the average cortical thickness and normalized volumes of some structures belonging to the same brain region.

The MTA score of each side was compared with the parahippocampal and entorhinal average cortical thicknesses or with hippocampal, parahippocampal, and entorhinal normalized volumes of the MTL.

The AT score of each side was compared with the average cortical thicknesses or the normalized volumes of the following temporal gyri (temporal pole, superior temporal, middle temporal, and inferior temporal gyri).

The PA score was compared with the average cortical thicknesses or the normalized volumes of the parietal lobes and cuneus gyrus. The OF score was compared with the average cortical thicknesses or the normalized volumes of the medial and lateral orbitofrontal gyri of both frontal lobes. The AC score was compared with the average cortical thicknesses or the normalized volumes of the rostral anterior cingulate and caudal anterior cingulate gyri of both frontal lobes.

The FI score of each side was compared with the average cortical thicknesses or the normalized volumes of the insula and pars opercularis, triangularis, and orbitalis of the frontal lobe.

Because the left and right hemispheres may show asymmetry in terms of atrophy, regional scores and thickness/volume measures were considered independently for each side for both semiquantitative and quantitative analysis.

### 2.7 Statistical analysis

Statistical analysis was conducted using Statistical Package for the Social Sciences (SPSS) for Windows version 24.0 (SPSS Inc., Chicago, IL, USA). Descriptive statistics were expressed as mean ± SD for continuous variables if not otherwise reported. For continuous variables, Shapiro–Wilk test was used to test the normality of data distribution.

Pearson correlation was used to assess the relationship between visual rating scores and the thickness/volume of corresponding brain regions.

Significant correlations of semiquantitative and quantitative measures were used to differentiate aMCI-AD from aMCI-S.

Kruskal–Wallis tests were performed to compare the semiquantitative (visual scores) and quantitative thickness/volume measurements among groups (aMCI-S, aMCI-AD, Controls) and *post-hoc* Mann–Whitney *U*-tests were performed between aMCI-S and aMCI-AD subgroups. Statistical significance was set at *P* < 0.05 and significance levels were adjusted according to the Bonferroni correction for multiple comparisons.

For visual rating scores and thickness/volume measures that resulted statistically different between the two aMCI subgroups (aMCI-S and aMCI-AD), receiver operating characteristic (ROC) curve analysis and area under the curves (AUC) were used to determine the optimal cut-off values capable of differentiating aMCI-AD from aMCI-S. Sensitivity and specificity were calculated for different cut-off points in the two aMCI groups. Combined visual rating scores and thickness/volume measures were included in the multivariate analysis in order to evaluate if the capability to differentiate aMCI-AD from aMCI-S improved. The multivariate analysis was conducted by developing, separately, logistic regression models for combined scores or thickness/volume measurements belonging to different brain atrophy regions. Thickness/volume measurements were normalized with the *z*-score before model fitting.

The ability of the models to discriminate between aMCI-S and aMCI-AD was evaluated by computing the AUC of the ROC curves. The best cut-off points were determined according to the Youden index method and used to compute sensitivity and specificity.

## 3 Results

### 3.1 Patient groups and clinical features

A total of 134 individuals were consecutively enrolled in the study. Based on the clinical evaluation performed after 2 years following up the diagnosis of aMCI, 54 patients were stable (aMCI-S) and 79 developed AD (aMCI-AD) (Table 1).

**Table 1:** Demographics and clinical data of aMCI-S and aMCI-AD patients at diagnosis

	aMCI-S (*n*. 54) (mean ± SD)	aMCI-AD (*n*. 79) (mean ± SD)
Age (years)	72.88 ± 6.81	72.19 ± 7.44
Gender	M = 25; F = 28	M = 39; F = 40
Educational level	12.20 ± 4.74	12.51 ± 4.24
MMSE	26.24 ± 2.52	26.11 ± 2.53
CDRS	0.5	0.5

MMSE, mini mental state examination; CDRS, clinical dementia rating scale; *n*, number; M, male; F, female; aMCI-S, stable amnesic MCI; aMCI-AD, progressive amnesic MCI.

Each subgroup was compared with 59 healthy control subjects matched for age and gender (age 72.23 + 3.55; M/F: 26/33) who performed brain MRI using the same scanner and protocol as patients with aMCI.

### 3.2 MRI evaluation

**Figure 2:**
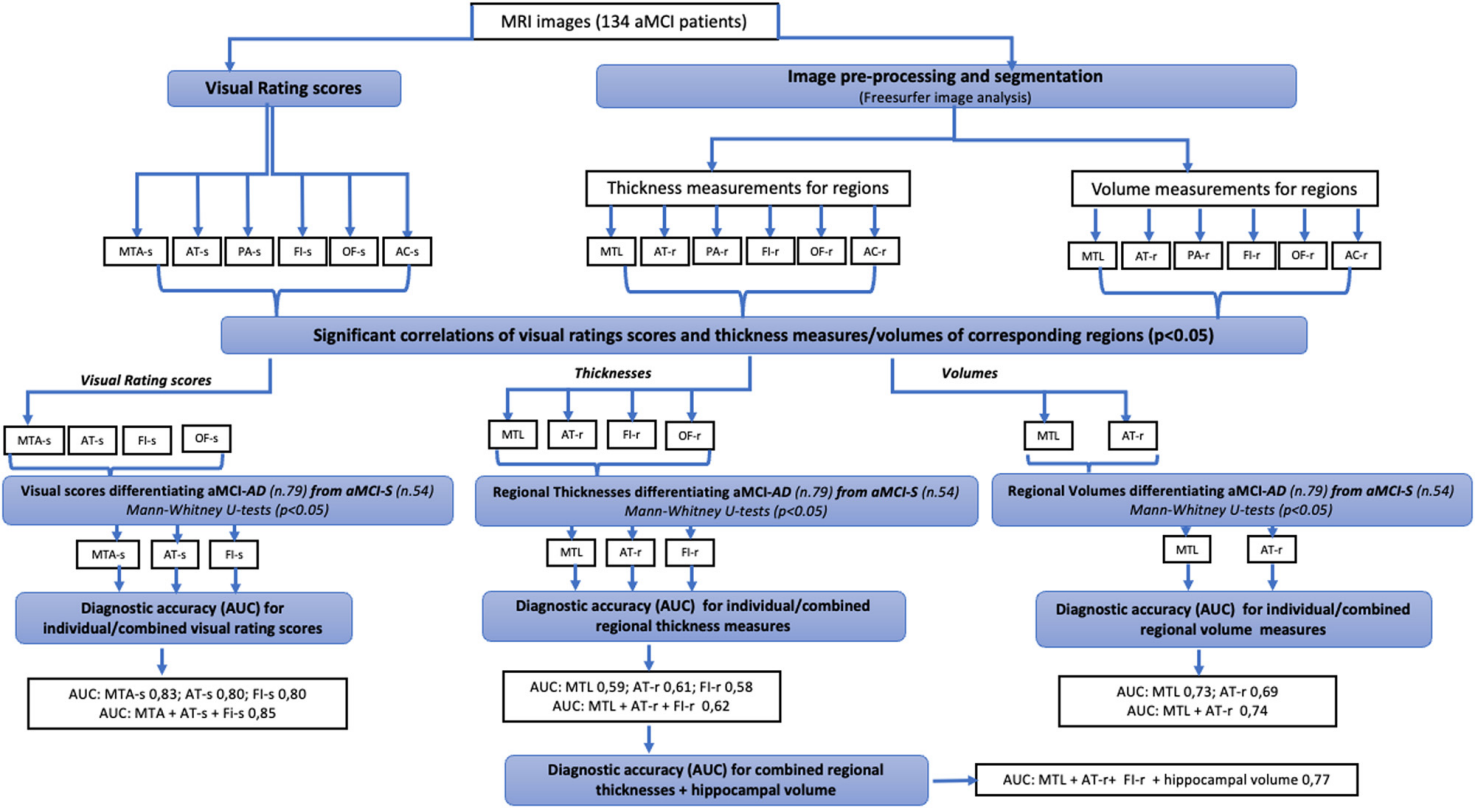
Workflow diagram showing the steps of MRI analysis. High resolution T1 MRI images of aMCI-S and aMCI-AD patients were used for quantitative and semiquantitative analysis. Correlations between semiquantitative visual ratings score and thickness measures/volumes of corresponding regions were used to assess the relationship between each visual rating scales and corresponding quantitative regional measures. Only the significant qualitative and quantitative measures (thicknesses and volumes) were used to differentiate aMCI-AD from aMCI-S. Only significant visual rating scores, cortical thickness, and volume measures between aMCI-S and aMCI-AD were used to calculate the diagnostic accuracy. Moreover, the multivariate analysis was conducted by logistic regression models for combined scores or thickness/volume measurements in order to evaluate if the capability in differentiating aMCI-AD from aMCI-S improved. AUC, area under the curve; MTA-s, medial temporal atrophy score; AT-s, anterior-temporal score; PA-s, posterior atrophy score; FI-s, fronto-insula score; OF-s, orbito-frontal score; AC-s, anterior cingulate score; MTL, medial temporal lobe; AT-r, anterior-temporal region; PA-r, posterior atrophy region; FI-r, fronto-insula region; OF-r, orbito-frontal region; AC-r, anterior cingulate region; and aMCI, amnesic mild cognitive impairment.

**Figure 3:**
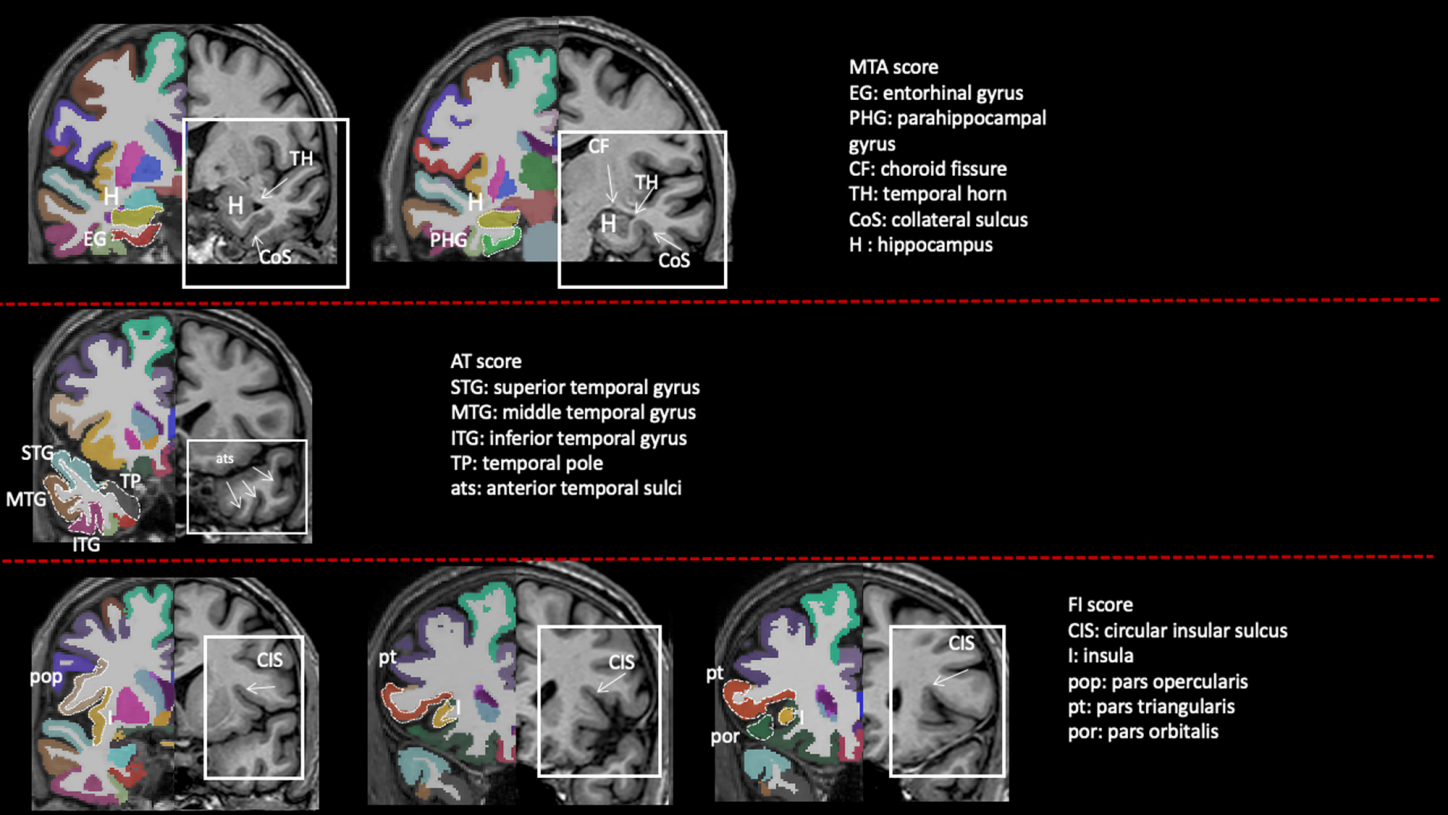
GM thickness and volume maps corresponding to brain regions evaluated by MTA, AT, and FI visual rating scales. MTA, AT, and FI visual rating scores are delimited by a white square while in the contralateral side white dotted line delimits some cortical gyri whose thickness or volume was assessed. MTA, medial temporal atrophy; AT, anterior temporal; and FI, fronto-insula.

At the time of diagnosis of aMCI (Time 0), all aMCI patients showed a negative correlation between MTA, AT, FI, OF visual rating scores and some GM thickness measures belonging to MTL, AT, FI, and OF regions. No correlation emerged between PA or AC visual rating scores and GM thickness measures within the same brain region. A negative correlation was found between MTA, AT visual rating scores and some GM volume measures belonging to MTL and AT regions. No correlation emerged between PA, FI, OF visual rating scores and GM volume measures within the same brain region (Table 2, Figures 2 and 3).

**Table 2:** Correlations of semiquantitative visual ratings score and thickness measures/volumes of corresponding regions in all aMCI patients at baseline

Visual rating score	Average cortical thicknesses	
MTA-s	Parahippocampal and entorhinal gyri	*P* < 0.001; *r*: −0.35
AT-s	Temporal pole, superior temporal, middle temporal, inferior temporal gyri	*P* < 0.001; *r*: −0.38
PA-s	Parietal gyri and cuneus gyrus	*P* = 0.12; *r*: −0.13
FI-s	Insula and frontal gyri (pars opercularis, triangularis, orbitalis)	*P* < 0.001; *r*: −0.27
OF-s	Medial and lateral orbitofrontal gyri	*P* = 0.001; *r*: −0.27
AC-s	Rostral and caudal anterior cingulate gyri	*P* = 0.88; *r*: −0.01

Visual rating score	Normalized volumes	
MTA-s	Hippocampal, parahippocampal, entorhinal gyri	*P* < 0.001; *r*: −0.20
AT-s	Temporal pole, superior temporal, middle temporal, inferior temporal gyri	*P* < 0.001; *r*: −0.27
PA-s	Parietal gyri and cuneus gyrus	*P* = 0.44; *r*: 0.06
FI-s	Insula and frontal gyri (pars opercularis, triangularis, orbitalis)	*P* = 0.62; *r*: −0.02
OF-s	Medial and lateral orbitofrontal gyri	*P* = 0.12; *r*: −0.13
AC-s	Rostral and caudal anterior cingulate gyri	*P* = 0.9; *r*: −0.28

MTA-s, middle temporal atrophy rating scale; AT-s, anterior temporal rating scale; PA-s, posterior atrophy rating scale; FI-s, fronto-insular rating scale; OF-s, orbito-frontal rating scale; AC-s, anterior cingulate rating scale.

**Figure 4:**
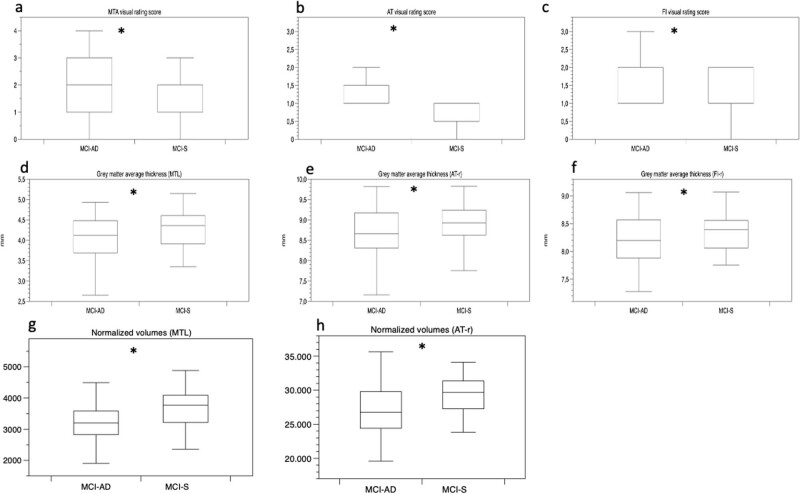
Whisker plots show MTA, AT, and FI visual rating scores (a–c), a set of matched cortical thickness measures (d–f), and normalized volumes (g, h) useful to differentiate aMCI-AD from aMCI-S. Plots show higher visual rating scores and lower GM thickness and volume measures in aMCI-AD than aMCI-S. The width of the plot shows the distribution of score values and thickness or volume measures for each corresponding rating scale. Whisker plots display the median values and IQR. Y-as: respectively, visual rating scores (a–c), GM average thickness (d–f), and GM normalized volumes (g, h) for aMCI-S and aMCI-AD groups. *Indicates significant difference between groups. MTA, medial temporal atrophy; AT, anterior temporal; FI, fronto-insula; MTL, medial temporal lobe; AT-r, anterior temporal region; FI-r, fronto-insula region; and aMCI, amnesic mild cognitive impairment.

Comparing aMCI subgroups (aMCI-S and aMCI-AD), a significant difference was found for MTA, AT, and FI rating scales, for cortical thickness belonging to MTL, AT, FI regions and for GM volumes belonging to MTL, AT regions with higher scores and lower thickness/volume measures in aMCI-AD than aMCI-S (Figures 2 and 4, Table 3).

**Table 3:** Cortical thicknesses and volume measures in aMCI-S, aMCI-AD, and control subjects

	aMCI-S median thickness [IQR] (mm)	aMCI-AD median thickness [IQR] (mm)	Controls median thickness [IQR] (mm)	*P*-value^**1**^aMCI-C	*P*-value^**2**^aMCI subgroups
Parahippocampal and entorhinal gyri (MTL)	4.358 [IQR 3.908–4.605]	4.122 [IQR 3.708–4.492]	4.215 [IQR 4.016–4.424]	0.003	0.002
Temporal pole, superior temporal, middle temporal, inferior temporal gyri (AT-r)	8.926 [IQR 8.628–9.241]	8.661 [IQR 8.31–9.196]	8.992 [IQR 8.652–9.169	0.001	0.001
Insula and frontal gyri (pars opercularis, triangularis, orbitalis) (FI-r)	8.389 [IQR 8.060–8.557]	8.194 [IQR 7.883–8.570]	8.286 [IQR 8.152–8.583]	0.037	0.044
Medial and lateral orbitofrontal gyri (OF-r)	8.369 [IQR 8.051–8.972]	8.300 [IQR 7.867–8.991]	8.106 [IQR 7.743–8.312]	0.003	0.957

	aMCI-S median volume[IQR] (mm^3^)	aMCI-AD median volume[IQR] (mm^3^)	Controls median thickness[IQR] (mm^3^)	*P*-value^**1**^aMCI -C	*P*-value^**2**^ aMCI subgroups
Hippocampal, parahippocampal and entorhinal gyri (MTL)	3,766.50 [IQR 3,215.002–4,087.003]	3,204.002 [IQR 2,829.002–3,598.002]	3,718.003 [IQR 3,493.003–3,938.001]	<0.001	<0.001
Temporal pole, superior temporal, middle temporal, inferior temporal gyri (AT-r)	29,670.502 [IQR 27,279.002–31,362.002]	26,780.002 [IQR 24,514.001–29,827.001]	29,852.502 [IQR 26,587.001–32,480.001]	<0.001	<0.001

Significant correlations of semiquantitative and quantitative measures shown in Table 2 were used to differentiate aMCI-AD from aMCI-S.

MTL, middle temporal lobe; AT-r, anterior temporal region; FI-r, fronto-insular region; OF-r, orbito-frontal region.

^**1**^Kruskal–Wallis test was performed among groups (aMCI-S, aMCI-AD, and Controls); ^**2**^ *post-hoc* Mann–Whitney *U*-test was performed between aMCI-S and aMCI-AD subgroups.

**Figure 5:**
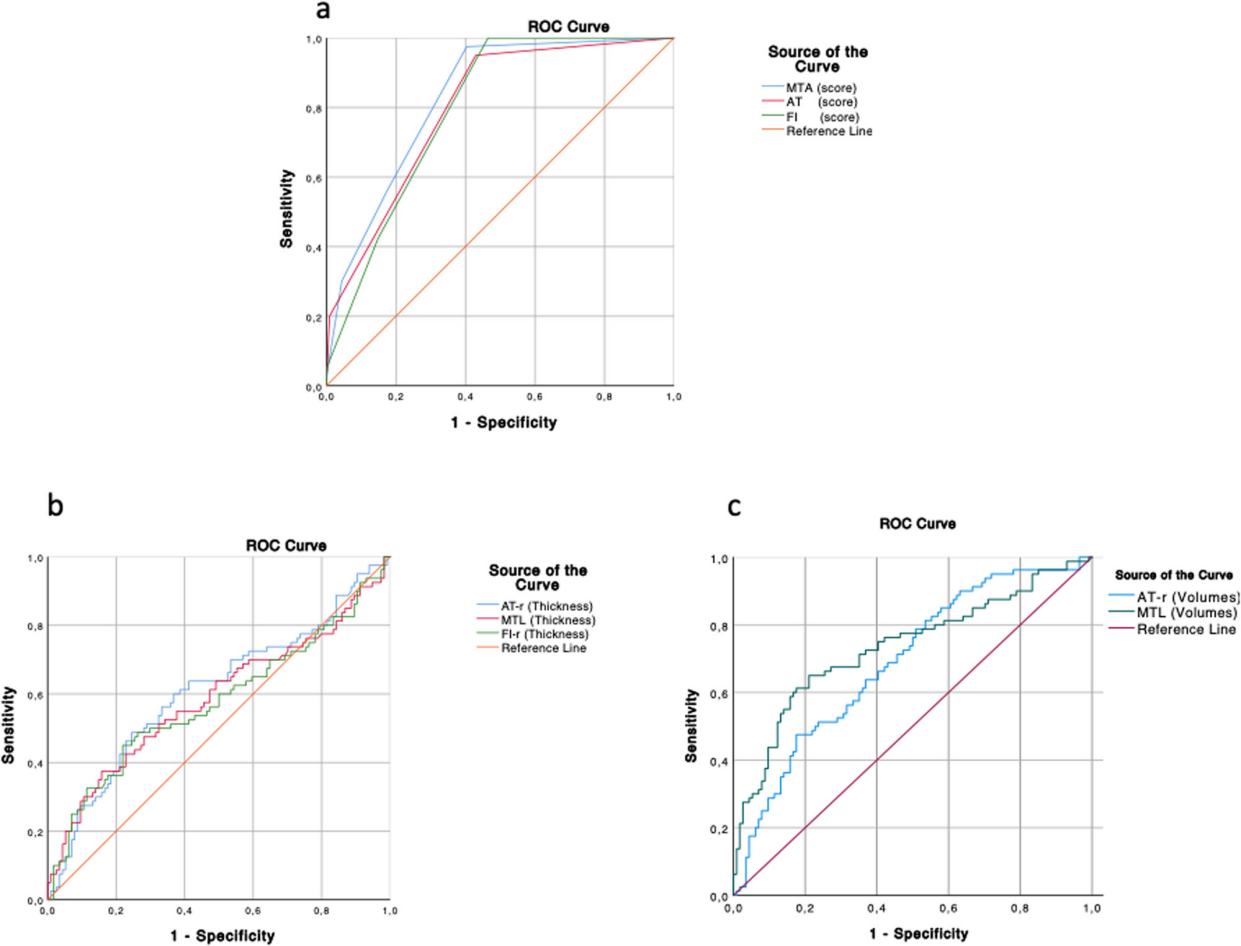
ROC curves of visual rating scales useful in differentiating aMCI-S from aMCI-AD (a). AUC = 0.83 for MTA score, 0.80 for AT score, 0.80 for FI score. ROC curves of a set of GM thickness measures belonging to brain regions assessed by visual rating scale, able to discriminate between aMCI-AD and aMCI-S (b). AUC = 0.59 for thickness measures of MTL, 0.61 for thickness measures of AT region, and 0.58 for thickness measures of FI region. ROC curves of a set of GM normalized volumes belonging to brain regions assessed by visual rating scale, able to discriminate between aMCI-AD and aMCI-S (c). AUC = 0.73 for normalized volumes of MTL, 0.69 for normalized volumes of AT region. AUC, area under the curve; MTA, medial temporal atrophy; AT, anterior-temporal; FI, fronto-insula; MTL, medial temporal lobe; AT-r, anterior-temporal region; FI-r, fronto-insula region; and aMCI, amnesic mild cognitive impairment.

ROC curve analysis using visual rating scores for MTA, AT, and FI showed a high diagnostic value (AUC: MTA-score 0.83, AT-score 0.80, FI-score 0.80) and, for AT score, the cut-off value >1.5 was able to differentiate MCI-S from MCI-AD with the best combination of sensitivity (80%) and specificity (99%) (Figures 2 and 5a, Table 4).

**Table 4:** Diagnostic accuracy of visual rating scales, regional cortical thickness, and volume measures in differentiating aMCI-AD from aMCI-S

	Cut-off value	AUC (95%CI)	Sen (%)	Spe (%)
Visual rating score				
MTA-s	>1.5	0.83 (0.79–0.87)	56	83
AT-s	>1.5	0.80 (0.75–0.84)	80	99
FI-s	>1.5	0.80 (0.75–0.84)	57	85
Average cortical thicknesses (mm)				
Parahippocampal and entorhinal gyri (MTL)	<4.24	0.59 (0.53–0.65)	60	53
Temporal pole, superior temporal, middle temporal, inferior temporal gyri (AT-r)	<8.88	0.61 (0.55–0.67)	61	59
Insula and frontal gyri (pars opercularis, triangularis, orbitalis) (FI-r)	<8.38	0.58 (0.52–0.64)	62.5	46
Normalized volumes (mm^3^)				
Hippocampal, parahippocampal and entorhinal gyri (MTL)	3,513,502	0.73 (0.68–0.79)	71	65
Temporal pole, superior temporal, middle temporal, inferior temporal gyri (AT-r)	28,423,001	0.69 (0.64–0.74)	66	60

The table considers only visual rating score, average cortical thickness, and normalized volume measures statistically different by comparing aMCI-S and aMCI-AD.

Sen, sensitivity; Spe, specificity; AUC, area under curve; CI, confidence interval.

MTA-s, middle temporal atrophy rating scale; AT-s, anterior temporal rating scale; FI-s, fronto-insular rating scale; MTL, middle temporal lobe; AT-r, anterior temporal region; FI-r, fronto-insular region.

ROC curve analysis for quantitative analysis showed that the diagnostic accuracy of thickness measures for MTL, AT, and FI regions was low (AUC: MTL 0.59, AT-region 0.61, FI-region 0.58) while the diagnostic accuracy of volume measures for MTL and AT regions was moderate (AUC: MTL 0.73, AT-r 0.69) (Figures 2 and 5b, c, Table 4).

The multivariate analysis for combined scores, thickness or/and volume measurements of matched brain atrophic regions demonstrated that the capability to differentiate aMCI-AD from aMCI-S improved only for visual rating scores (AUC 0.85; sensitivity 95%) or for thickness measures along with hippocampal volume (AUC 0.77; sensitivity 76%). In the first case, the AT score showed a greater influence compared to the MTA and FI scores given by its higher model coefficient (1.77). In the second case, the hippocampal volume provided a greater influence compared to the thickness measures given by its higher model coefficient (−1.13).

On the other hand, considering only combined thickness measures, diagnostic value and sensitivity were comparable to those of the single region (AUC 0.62; sensitivity 57%), while considering only combined volume measures, a comparable diagnostic value emerged (AUC 0.74) but with a lower sensitivity (61%) than to those of the single region (Figure 2, Table 5).

**Table 5:** Logistic regression model developed by combining visual rating scores and regional thickness/volume measures in differentiating aMCI-AD from aMCI-S

	AUC (95% CI)	Sen (%)	Spec (%)
Visual rating score/subscore			
MTA-s + AT-s + FI-s	0.85 (0.81–0.88)	95	64
Average cortical thicknesses (mm)			
Parahippocampal and entorhinal gyri (MTL) + temporal pole, superior temporal, middle temporal, inferior temporal gyri (AT-r) + insula, frontal gyri (pars opercularis, triangularis, orbitalis) (FI-r)	0.62 (0.55–0.68)	57	75
Normalized volumes (mm^3^)			
Hippocampal, parahippocampal and entorhinal gyri (MTL) + temporal pole, superior temporal, middle temporal, inferior temporal gyri (AT-r)	0.74 (0.69–0.79)	61	77
Average cortical thicknesses (mm) + normalized hippocampal volume (mm^3^)			
Parahippocampal and entorhinal gyri thicknesses (MTL) + temporal pole, superior temporal, middle temporal, inferior temporal gyri thicknesses (AT-r) + insula, frontal gyri thicknesses (pars opercularis, triangularis, orbitalis) (FI-r) + hippocampal volume	0.77 (0.72–0.81)	76	67

Sen, sensitivity; Spec, specificity; AUC, area under curve, CI, confidence interval.

MTA-s, middle temporal atrophy rating scale; AT-s, anterior temporal rating scale; FI-s, fronto-insular rating scale; MTL, middle temporal lobe; AT-r, anterior temporal region; FI-r, fronto-insular region.

## 4 Discussion

Structural MRI is a leading diagnostic tool for patients with cognitive impairment [16,21], but its usefulness in differentiating patients at the preclinical stage of AD is limited because of patients heterogeneity and observers’ experience, and it requires several post-processing software [51,52].

Previous studies, performed using 1.5T MRI, demonstrated that cortical thinning and GM volume reduction begin mainly from the region of MTL and spread up to temporal, frontal, and parietal areas as the disease progresses but the structural changes of aMCI pathology are heterogeneous [53,54]. Other studies showed that 3T MRI is more informative and potentially more suitable for the parcellation of the cerebral cortex and the segmentation of the subcortical structures in the brain because of a higher signal-to-noise ratio than 1.5T MRI [55]. However, in clinical practice, the theoretical doubling of the signal-to-noise ratio is only 25%, and 1.5T and 3T scans do not significantly differ in their power to detect differences in quantitative values [56].

To date, structural MRI studies in aMCI patients have been mainly focused on the subregions of the MTL but other studies demonstrated that a combination of different structural measures within the frontotemporal network might be more accurate than measures of single structures for predicting future conversion from MCI to AD [57–59]. Although some studies focused on measures of the brain’s region volume, these kinds of measures are biased by some anatomical factors which may vary among individuals, such as the depth of the collateral sulcus [50,60–62]. Conversely, thickness measures have not been fully evaluated at the early stage of disease, although the cytoarchitectural structure of the GM shows a lower variability [51] and cortical thinning is more prominent than volume changes [22]. Therefore, thickness evaluation of specific brain regions identified by visual scales may be a promising tool to objectively quantify the global and regional atrophy at the early stage of the disease.

The first goal of this study was to explore, in aMCI patients at an early stage, the relationship between validated visual rating scales and some thickness or volume measurements belonging to the same regions in order to assess the agreement between regional quantitative and visual semiquantitative analyses.

Our data showed that, at the prodromal stage of aMCI, quantitative measures might reveal the atrophic pattern detected by visual scales in frontotemporal regions. This finding suggests a specific regional atrophic pattern; in particular, higher rating scores were associated with lower GM thickness/volume regional measures. Moreover, our data demonstrated that, at the early stage of MCI, visual scales matched more with thickness measures than with volume measures because the early loss of GM is better evaluated by thickness measures [22] that may detect changes also in other cortical regions, beyond MTA and AT regions.

The second aim was to assess the accuracy of visual rating scores and quantitative analyses in differentiating aMCI-S and aMCI-AD in the short term. Our analysis showed that MTA, AT, and FI visual rating scores besides a set of thickness measurements within the MTL, AT, and FI regions and a set of volume measurements within the MTL and AT regions were significantly different between aMCI-S and aMCI-AD. These data show that, at the early stage of disease, selected visual rating scores and a set of thickness/volume measures in specific regions of frontotemporal network may discriminate aMCI-AD from aMCI-S patients because aMCI-AD showed higher scores and lower cortical thickness/GM volumes than aMCI-S.

The diagnostic accuracy of semiquantitative analysis was higher, in particular for MTA, AT, and FI visual rating scales (AUC: MTA-score 0.83, AT-score 0.80, and FI-score 0.80). The cut-off value >1.5 of AT visual rating scale was able to differentiate aMCI-S by aMCI-AD with the best combination of sensitivity (80%) and specificity (99%). It is conceivable that early changes in AT region are determined by better visibility of the CSF/cortex interface due to its wider dimensions.

On the other hand, we found that the diagnostic accuracy of some volume measures in MTL and AT regions was moderate (AUC: MTL 0.73 and AT-region 0.69), whereas the diagnostic accuracy of some thickness measures in MTL, AT, and FI regions was low (AUC: MTL 0.59, AT-region 0.61, and FI-region 0.58). The reason for the better accuracy of volume measures compared to thickness measures may be related to the inclusion of hippocampal structures only in volume measures; in fact, the hippocampus is a subcortical structure [63], and it is the main involved structure in AD [8,28].

Thus, in agreement with previous studies, our data confirm that expert semiquantitative analysis is more suitable for differentiating aMCI-S and aMCI-AD subgroups when compared to quantitative structural measures because the neuroradiological regional semiquantitative visual inspection can better catch the spatial-temporal pattern of brain atrophy [64]. In fact, the standardized visual rating scales, assessing the relationship between brain structures and liquor spaces of sectorized regions, provide a more complete picture of regional atrophy patterns.

The last goal was to evaluate whether the combination of some visual rating scales and corresponding quantitative measures improved the accuracy in differentiating aMCI-AD from aMCI-S. To this aim, we developed a model of combined visual rating scores, thickness and or volume measures. This analysis showed an improvement in the capability to differentiate aMCI-AD from aMCI-S when regional thickness measures along with the hippocampal volume were combined. This was mostly due to the greater influence of the hippocampal volume compared to the thickness measures in the combined model, which can be explained by the key role of the hippocampus in AD.

Although combined regional thickness measures with hippocampal volume have better diagnostic accuracy than the combined regional thickness or volume measurements alone, visual rating scores showed even better results when assessed by expert observers, with a greater influence of the AT score in the model combining different visual scores. Thus, semiquantitative findings may be considered as surrogate parameters helpful for differentiating aMCI-AD from aMCI-S and their diagnostic accuracy may be increased when visual rating scores of specific regions are combined.

This study has some limitations. First, the study’s results may be influenced by the retrospective design. Second, semiquantitative and quantitative analyses of brain MR were done using a 1.5T MRI. However, our results are helpful in proposing prospective semiquantitative brain MR studies performed by experienced and moderately experienced observers trained in this scoring system to evaluate the impact of experience in this kind of evaluation. Longitudinal quantitative studies assessing differences in thickness and volume measures between 1.5T and 3T MRI scanners in aMCI patients could help to understand if MRI with higher field strength provides more advantages in detecting earlier signs of brain atrophy.

## 5 Conclusion

Our study demonstrated that visual rating scales, assessed by experienced neuroradiologists, has better accuracy than quantitative measures of corresponding brain structures in detecting atrophy in specific brain regions, even when thickness measurements were combined to hippocampal volume.

Semiquantitative evaluation, performed at the early stage of disease by expert observers, may be a fast and reliable diagnostic tool capable of differentiating aMCI patients that may evolve to AD, because they may catch both hippocampal volume loss and early cortical thickness changes.

## References

[1] Ferri CP, Prince M, Brayne C, Brodaty H, Fratiglioni L, Ganguli M, et al. Global prevalence of dementia: a Delphi consensus study. Lancet Lond Engl. 2005;366(9503):2112–7. 10.1016/S0140-6736(05)67889-0 PMC2850264 16360788

[2] Farias ST, Mungas D, Reed BR, Harvey D, DeCarli C. Progression of mild cognitive impairment to dementia in clinic- vs community-based cohorts. Arch Neurol. 2009;66(9):1151–7. 10.1001/archneurol.2009.106 PMC2863139 19752306

[3] Petersen RC. Mild cognitive impairment as a diagnostic entity. J Intern Med. 2004;256(3):183–94. 10.1111/j.1365-2796.2004.01388.x 15324362

[4] Mitchell J, Arnold R, Dawson K, Nestor PJ, Hodges JR. Outcome in subgroups of mild cognitive impairment (MCI) is highly predictable using a simple algorithm. J Neurol. 2009;256(9):1500–9. 10.1007/s00415-009-5152-0 19434441

[5] Petersen RC. Clinical practice. Mild cognitive impairment. N Engl J Med. 2011;364(23):2227–34. 10.1056/NEJMcp0910237 21651394

[6] Ewers M, Sperling RA, Klunk WE, Weiner MW, Hampel H. Neuroimaging markers for the prediction and early diagnosis of Alzheimer’s disease dementia. Trends Neurosci. 2011;34(8):430–42. 10.1016/j.tins.2011.05.005 PMC3275347 21696834

[7] Falahati F, Ferreira D, Muehlboeck JS, Eriksdotter M, Simmons A, Wahlund LO, et al. Monitoring disease progression in mild cognitive impairment: associations between atrophy patterns, cognition, APOE and amyloid. NeuroImage Clin. 2017;16:418–28. 10.1016/j.nicl.2017.08.014 PMC5573795 28879083

[8] Dubois B, Feldman HH, Jacova C, Dekosky ST, Barberger-Gateau P, Cummings J, et al. Research criteria for the diagnosis of Alzheimer’s disease: revising the NINCDS-ADRDA criteria. Lancet Neurol. 2007;6(8):734–46. 10.1016/S1474-4422(07)70178-3 17616482

[9] Dubois B, Feldman HH, Jacova C, Cummings JL, Dekosky ST, Barberger-Gateau P, et al. Revising the definition of Alzheimer’s disease: a new lexicon. Lancet Neurol. 2010;9(11):1118–27. 10.1016/S1474-4422(10)70223-4 20934914

[10] Albert MS, DeKosky ST, Dickson D, Dubois B, Feldman HH, Fox NC, et al. The diagnosis of mild cognitive impairment due to Alzheimer’s disease: recommendations from the National Institute on Aging-Alzheimer’s Association workgroups on diagnostic guidelines for Alzheimer’s disease. Alzheimers Dement J Alzheimers Assoc. 2011;7(3):270–9. 10.1016/j.jalz.2011.03.008 PMC3312027 21514249

[11] Pilato F, Profice P, Ranieri F, Capone F, Di Iorio R, Florio L, et al. Synaptic plasticity in neurodegenerative diseases evaluated and modulated by in vivo neurophysiological techniques. Mol Neurobiol. 2012;46(3):563–71. 10.1007/s12035-012-8302-9 22821187

[12] Popuri K, Ma D, Wang L, Beg MF. Using machine learning to quantify structural MRI neurodegeneration patterns of Alzheimer’s disease into dementia score: independent validation on 8,834 images from ADNI, AIBL, OASIS, and MIRIAD databases. Hum Brain Mapp. 2020;41(14):4127–47. 10.1002/hbm.25115 PMC7469784 32614505

[13] Jack CR Jr, Shiung MM, Weigand SD, O'brien PC, Gunter JL, Boeve BF, et al. Brain atrophy rates predict subsequent clinical conversion in normal elderly and amnestic MCI. Neurology. 2005;65(8):1227–31. 10.1212/01.wnl.0000180958.22678.91 PMC2753547 16247049

[14] Park SW, Kim S, Park J, Jang JW, Kim S. A comprehensive visual rating scale for predicting progression from mild cognitive impairment to dementia in patients with Alzheimer’s pathology or suspected non-Alzheimer’s pathology. Dement Neurocognitive Disord. 2020;19(4):129–39. 10.12779/dnd.2020.19.4.129 PMC7781734 33377666

[15] Zimny A, Bladowska J, Neska M, Petryszyn K, Guziński M, Szewczyk P, et al. Quantitative MR evaluation of atrophy, as well as perfusion and diffusion alterations within hippocampi in patients with Alzheimer’s disease and mild cognitive impairment. Med Sci Monit Int Med J Exp Clin Res. 2013;19:86–94. 10.12659/MSM.883757 PMC3628917 23377218

[16] Harper L, Fumagalli GG, Barkhof F, Scheltens P, O’Brien JT, Bouwman F, et al. MRI visual rating scales in the diagnosis of dementia: evaluation in 184 post-mortem confirmed cases. Brain J Neurol. 2016;139(Pt 4):1211–25. 10.1093/brain/aww005 PMC4806219 26936938

[17] Harper L, Barkhof F, Fox NC, Schott JM. Using visual rating to diagnose dementia: a critical evaluation of MRI atrophy scales. J Neurol Neurosurg Psychiatry. 2015;86(11):1225–33. 10.1136/jnnp-2014-310090 25872513

[18] Scheltens P, Leys D, Barkhof F, Huglo D, Weinstein HC, Vermersch P, et al. Atrophy of medial temporal lobes on MRI in “probable” Alzheimer’s disease and normal ageing: diagnostic value and neuropsychological correlates. J Neurol Neurosurg Psychiatry. 1992;55(10):967–72. 10.1136/jnnp.55.10.967 PMC1015202 1431963

[19] Scheltens P, Launer LJ, Barkhof F, Weinstein HC, van Gool WA. Visual assessment of medial temporal lobe atrophy on magnetic resonance imaging: interobserver reliability. J Neurol. 1995;242(9):557–60. 10.1007/BF00868807 8551316

[20] Dale AM, Fischl B, Sereno MI. Cortical surface-based analysis. I. Segmentation and surface reconstruction. Neuroimage. 1999;9(2):179–94. 10.1006/nimg.1998.0395 9931268

[21] Traschütz A, Enkirch SJ, Polomac N, Widmann CN, Schild HH, Heneka MT, et al. The entorhinal cortex atrophy score is diagnostic and prognostic in mild cognitive impairment. J Alzheimers Dis. 2020;75(1):99–108. 10.3233/JAD-181150 32250289

[22] Ma X, Li Z, Jing B, Liu H, Li D, Li H, et al. Identify the atrophy of Alzheimer’s disease, mild cognitive impairment and normal aging using morphometric MRI analysis. Front Aging Neurosci. 2016;8:243. 10.3389/fnagi.2016.00243 PMC5067377 27803665

[23] Xie L, Wisse LEM, Pluta J, de Flores R, Piskin V, Manjón JV, et al. Automated segmentation of medial temporal lobe subregions on in vivo T1-weighted MRI in early stages of Alzheimer’s disease. Hum Brain Mapp. 2019;40(12):3431–51. 10.1002/hbm.24607 PMC6697377 31034738

[24] Liu Y, Paajanen T, Zhang Y, Westman E, Wahlund LO, Simmons A, et al. Combination analysis of neuropsychological tests and structural MRI measures in differentiating AD, MCI and control groups – the AddNeuroMed study. Neurobiol Aging. 2011;32(7):1198–206. 10.1016/j.neurobiolaging.2009.07.008 19683363

[25] Jeong HE, Shin DH, Lee DC. Medial temporal atrophy alone is insufficient to predict underlying Alzheimer’s disease pathology. Korean J Fam Med. 2020;41(5):352–8. 10.4082/kjfm.18.0144 PMC7509126 32521990

[26] Atiya M, Hyman BT, Albert MS, Killiany R. Structural magnetic resonance imaging in established and prodromal Alzheimer disease: a review. Alzheimer Dis Assoc Disord. 2003;17(3):177–95. 10.1097/00002093-200307000-00010 14512832

[27] Winblad B, Palmer K, Kivipelto M, Jelic V, Fratiglioni L, Wahlund LO, et al. Mild cognitive impairment – beyond controversies, towards a consensus: report of the international working group on mild cognitive impairment. J Intern Med. 2004;256(3):240–6. 10.1111/j.1365-2796.2004.01380.x 15324367

[28] McKhann GM, Knopman DS, Chertkow H, Hyman BT, Jack CR, Kawas CH, et al. The diagnosis of dementia due to Alzheimer’s disease: recommendations from the National Institute on Aging-Alzheimer’s Association workgroups on diagnostic guidelines for Alzheimer’s disease. Alzheimers Dement J Alzheimers Assoc. 2011;7(3):263–9. 10.1016/j.jalz.2011.03.005 PMC3312024 21514250

[29] Oldfield RC. The assessment and analysis of handedness: the Edinburgh inventory. Neuropsychologia. 1971;9(1):97–113. 10.1016/0028-3932(71)90067-4 5146491

[30] Falgàs N, Balasa M, Bargalló N, Borrego-Écija S, Ramos-Campoy O, Fernández-Villullas G, et al. Diagnostic accuracy of MRI visual rating scales in the diagnosis of early onset cognitive impairment. J Alzheimers Dis. 2020;73(4):1575–83. 10.3233/JAD-191167 31958089

[31] Fumagalli GG, Basilico P, Arighi A, Bocchetta M, Dick KM, Cash DM, et al. Distinct patterns of brain atrophy in Genetic Frontotemporal Dementia Initiative (GENFI) cohort revealed by visual rating scales. Alzheimers Res Ther. 2018;10(1):46. 10.1186/s13195-018-0376-9 PMC5968621 29793546

[32] Koedam EL, Lehmann M, van der Flier WM, Scheltens P, Pijnenburg YA, Fox N, et al. Visual assessment of posterior atrophy development of a MRI rating scale. Eur Radiol. 2011;21(12):2618–25. 10.1007/s00330-011-2205-4 PMC3217148 21805370

[33] Davies RR, Kipps CM, Mitchell J, Kril JJ, Halliday GM, Hodges JR. Progression in frontotemporal dementia: identifying a benign behavioral variant by magnetic resonance imaging. Arch Neurol. 2006;63(11):1627–31. 10.1001/archneur.63.11.1627 17101833

[34] Kipps CM, Davies RR, Mitchell J, Kril JJ, Halliday GM, Hodges JR. Clinical significance of lobar atrophy in frontotemporal dementia: application of an MRI visual rating scale. Dement Geriatr Cogn Disord. 2007;23(5):334–42. 10.1159/000100973 17374952

[35] Davies RR, Scahill VL, Graham A, Williams GB, Graham KS, Hodges JR. Development of an MRI rating scale for multiple brain regions: comparison with volumetrics and with voxel-based morphometry. Neuroradiology. 2009;51(8):491–503. 10.1007/s00234-009-0521-z 19308367

[36] Hornberger M, Savage S, Hsieh S, Mioshi E, Piguet O, Hodges JR. Orbitofrontal dysfunction discriminates behavioral variant frontotemporal dementia from Alzheimer’s disease. Dement Geriatr Cogn Disord. 2010;30(6):547–52. 10.1159/000321670 21252550

[37] Ambikairajah A, Devenney E, Flanagan E, Yew B, Mioshi E, Kiernan MC, et al. A visual MRI atrophy rating scale for the amyotrophic lateral sclerosis-frontotemporal dementia continuum. Amyotroph Lateral Scler Front Degener. 2014;15(3–4):226–34. 10.3109/21678421.2014.880180 24533506

[38] Chepkoech JL, Walhovd KB, Grydeland H, Fjell AM. Alzheimer’s disease neuroimaging initiative. Effects of change in FreeSurfer version on classification accuracy of patients with Alzheimer’s disease and mild cognitive impairment. Hum Brain Mapp. 2016;37(5):1831–41. 10.1002/hbm.23139 PMC6867543 27018380

[39] Ségonne F, Dale AM, Busa E, Glessner M, Salat D, Hahn HK, et al. A hybrid approach to the skull stripping problem in MRI. Neuroimage. 2004;22(3):1060–75. 10.1016/j.neuroimage.2004.03.032 15219578

[40] Fischl B, Salat DH, Busa E, Albert M, Dieterich M, Haselgrove C, et al. Whole brain segmentation: automated labeling of neuroanatomical structures in the human brain. Neuron. 2002;33(3):341–55. 10.1016/s0896-6273(02)00569-x 11832223

[41] Desikan RS, Ségonne F, Fischl B, Quinn BT, Dickerson BC, Blacker D, et al. An automated labeling system for subdividing the human cerebral cortex on MRI scans into gyral based regions of interest. NeuroImage. 2006;31(3):968–80. 10.1016/j.neuroimage.2006.01.021 16530430

[42] Reuter M, Rosas HD, Fischl B. Highly accurate inverse consistent registration: a robust approach. Neuroimage. 2010;53(4):1181–96. 10.1016/j.neuroimage.2010.07.020 PMC2946852 20637289

[43] Fischl B, Salat DH, van der Kouwe AJ, Makris N, Ségonne F, Quinn BT, et al. Sequence-independent segmentation of magnetic resonance images, sequence-independent segmentation of magnetic resonance images. NeuroImage. 2004;23 Suppl 1:S69–84. 10.1016/j.neuroimage.2004.07.016 15501102

[44] Sled JG, Zijdenbos AP, Evans AC. A nonparametric method for automatic correction of intensity nonuniformity in MRI data. IEEE Trans Med Imaging. 1998;17(1):87–97. 10.1109/42.668698 9617910

[45] Fischl B, Liu A, Dale AM. Automated manifold surgery: constructing geometrically accurate and topologically correct models of the human cerebral cortex. IEEE Trans Med Imaging. 2001;20(1):70–80. 10.1109/42.906426 11293693

[46] Segonne F, Pacheco J, Fischl B. Geometrically accurate topology-correction of cortical surfaces using nonseparating loops. IEEE Trans Med Imaging. 2007;26(4):518–29. 10.1109/TMI.2006.887364 17427739

[47] Dale AM, Sereno MI. Improved localizadon of cortical activity by combining EEG and MEG with MRI cortical surface reconstruction: a linear approach. J Cogn Neurosci Spring. 1993;5(2):162–76. 10.1162/jocn.1993.5.2.162 23972151

[48] Fischl B, Dale AM. Measuring the thickness of the human cerebral cortex from magnetic resonance images. Proc Natl Acad Sci USA. 2000;97(20):11050–5. 10.1073/pnas.200033797 PMC27146 10984517

[49] Desikan RS, Ségonne F, Fischl B, Quinn BT, Dickerson BC, Blacker D, et al. An automated labeling system for subdividing the human cerebral cortex on MRI scans into gyral based regions of interest. Neuroimage. 2006;31(3):968–80. 10.1016/j.neuroimage.2006.01.021 16530430

[50] Feczko E, Augustinack JC, Fischl B, Dickerson BC. An MRI-based method for measuring volume, thickness and surface area of entorhinal, perirhinal, and posterior parahippocampal cortex. Neurobiol Aging. 2009;30(3):420–31. 10.1016/j.neurobiolaging.2007.07.023 PMC3665765 17850926

[51] Julkunen V, Niskanen E, Muehlboeck S, Pihlajamäki M, Könönen M, Hallikainen M, et al. Cortical thickness analysis to detect progressive mild cognitive impairment: a reference to Alzheimer’s disease. Dement Geriatr Cogn Disord. 2009;28(5):404–12. 10.1159/000256274 19907176

[52] Brewer JB, Magda S, Airriess C, Smith ME. Fully-automated quantification of regional brain volumes for improved detection of focal atrophy in Alzheimer disease. AJNR Am J Neuroradiol. 2009;30(3):578–80. 10.3174/ajnr.A1402 PMC5947999 19112065

[53] Singh V, Chertkow H, Lerch JP, Evans AC, Dorr AE, Kabani NJ. Spatial patterns of cortical thinning in mild cognitive impairment and Alzheimer’s disease. Brain J Neurol. 2006;129(Pt 11):2885–93. 10.1093/brain/awl256 17008332

[54] McDonald CR, McEvoy LK, Gharapetian L, Fennema-Notestine C, Hagler DJ Jr, Holland D, et al. Regional rates of neocortical atrophy from normal aging to early Alzheimer disease. Neurology. 2009;73(6):457–65. 10.1212/WNL.0b013e3181b16431 PMC2727145 19667321

[55] Heinen R, Bouvy WH, Mendrik AM, Viergever MA, Biessels GJ, de Bresser J. Robustness of automated methods for brain volume measurements across different MRI field strengths. PLoS One. 2016;11(10):e0165719. 10.1371/journal.pone.0165719 PMC5087903 27798694

[56] Wardlaw JM, Brindle W, Casado AM, Shuler K, Henderson M, Thomas B, et al. A systematic review of the utility of 1.5 versus 3 Tesla magnetic resonance brain imaging in clinical practice and research. Eur Radiol. 2012;22(11):2295–303. 10.1007/s00330-012-2500-8 22684343

[57] Klöppel S, Stonnington CM, Chu C, Draganski B, Scahill RI, Rohrer JD, et al. Automatic classification of MR scans in Alzheimer’s disease. Brain J Neurol. 2008;131(Pt 3):681–9. 10.1093/brain/awm319 PMC2579744 18202106

[58] Westman E, Aguilar C, Muehlboeck JS, Simmons A. Regional magnetic resonance imaging measures for multivariate analysis in Alzheimer’s disease and mild cognitive impairment. Brain Topogr. 2013;26(1):9–23. 10.1007/s10548-012-0246-x PMC3536978 22890700

[59] Zhang D, Wang Y, Zhou L, Yuan H, Shen D. Alzheimer’s disease neuroimaging initiative. Multimodal classification of Alzheimer’s disease and mild cognitive impairment. NeuroImage. 2011;55(3):856–67. 10.1016/j.neuroimage.2011.01.008 PMC3057360 21236349

[60] Schwarz CG, Gunter JL, Wiste HJ, Przybelski SA, Weigand SD, Ward CP, et al. A large-scale comparison of cortical thickness and volume methods for measuring Alzheimer’s disease severity. NeuroImage Clin. 2016;11:802–12. 10.1016/j.nicl.2016.05.017 PMC5187496 28050342

[61] Berron D, Vieweg P, Hochkeppler A, Pluta JB, Ding SL, Maass A, et al. A protocol for manual segmentation of medial temporal lobe subregions in 7 Tesla MRI. NeuroImage Clin. 2017;15:466–82. 10.1016/j.nicl.2017.05.022 PMC5476466 28652965

[62] Chauveau L, Kuhn E, Palix C, Felisatti F, Ourry V, de La Sayette V, et al. Medial temporal lobe subregional atrophy in aging and Alzheimer’s disease: a longitudinal study. Front Aging Neurosci. 2021;13:750154. 10.3389/fnagi.2021.750154 PMC8554299 34720998

[63] Um YH, Wang SM, Kang DW, Kim NY, Lim HK. Subcortical and cerebellar neural correlates of prodromal Alzheimer’s disease with prolonged sleep latency. J Alzheimers Dis JAD. 2022;86:565–78. 10.3233/JAD-215460 PMC9028620 35068468

[64] Misra C, Fan Y, Davatzikos C. Baseline and longitudinal patterns of brain atrophy in MCI patients, and their use in prediction of short-term conversion to AD: results from ADNI. NeuroImage. 2009;44(4):1415–22. 10.1016/j.neuroimage.2008.10.031 PMC2648825 19027862

